# ExacTrac Dynamic workflow evaluation: Combined surface optical/thermal imaging and X‐ray positioning

**DOI:** 10.1002/acm2.13754

**Published:** 2022-08-24

**Authors:** Vanessa Da Silva Mendes, Michael Reiner, Lili Huang, Daniel Reitz, Katrin Straub, Stefanie Corradini, Maximilian Niyazi, Claus Belka, Christopher Kurz, Guillaume Landry, Philipp Freislederer

**Affiliations:** ^1^ Department of Radiation Oncology University Hospital LMU Munich Munich Germany; ^2^ German Cancer Consortium (DKTK) Partner Site Munich Munich Germany

**Keywords:** IGRT, optical surface imaging, SGRT, stereoscopic X‐ray imaging, X‐ray intra‐fractional monitoring

## Abstract

In modern radiotherapy (RT), especially for stereotactic radiotherapy or stereotactic radiosurgery treatments, image guidance is essential. Recently, the ExacTrac Dynamic (EXTD) system, a new combined surface‐guided RT and image‐guided RT (IGRT) system for patient positioning, monitoring, and tumor targeting, was introduced in clinical practice. The purpose of this study was to provide more information about the geometric accuracy of EXTD and its workflow in a clinical environment. The surface optical/thermal‐ and the stereoscopic X‐ray imaging positioning systems of EXTD was evaluated and compared to cone‐beam computed tomography (CBCT). Additionally, the congruence with the radiation isocenter was tested. A Winston Lutz test was executed several times over 1 year, and repeated end‐to‐end positioning tests were performed. The magnitude of the displacements between all systems, CBCT, stereoscopic X‐ray, optical‐surface imaging, and MV portal imaging was within the submillimeter range, suggesting that the image guidance provided by EXTD is accurate at any couch angle. Additionally, results from the evaluation of 14 patients with intracranial tumors treated with open‐face masks are reported, and limited differences with a maximum of 0.02 mm between optical/thermal‐ and stereoscopic X‐ray imaging were found. As the optical/thermal positioning system showed a comparable accuracy to other IGRT systems, and due to its constant monitoring capability, it can be an efficient tool for detecting intra‐fractional motion and for real‐time tracking of the surface position during RT.

## 1 INTRODUCTION

Accurate tumor localization and patient setup are essential for precise external beam radiotherapy.^1^ Moreover, real‐time patient monitoring throughout all treatment fractions enables not only the detection of inter‐ and intra‐fractional anatomical variations, but also immediate correction of the target position or the possibility of replanning.^2^, ^3^ These aspects are critical for hypo‐fractionated treatments.^4^ Image‐guided radiotherapy (IGRT) can make conventional radiotherapy (RT) safer by virtue of increased precision of delivery. In addition, it facilitates the application of specialized irradiation techniques with narrow safety margins, reducing the probability of adverse effects, and is therefore an essential component in modern RT.^3^

Linear accelerator (linac)‐based stereotactic radiotherapy (SRT) and stereotactic radiosurgery (SRS) cone‐beam computed tomography (CBCT) or stereoscopic X‐ray imaging, such as with the ExacTrac system (Brainlab AG, Munich, Germany), are both commonly used for IGRT.^5^ CBCT enables three‐dimensional (3D) volumetric imaging, which provides better visualization of the internal anatomy and more information for image registration in comparison to stereoscopic X‐ray imaging.^5^ Compared to CBCT, the ExacTrac stereoscopic X‐ray system provides faster imaging,^5^ lower dose exposure,^6^, ^7^ and image guidance for noncoplanar treatments.^5^ The use of surface guidance in RT has also been widely implemented for patient positioning, increasing patient setup information compared to laser‐based setup.^8^ Surface‐guided RT (SGRT) may improve not only patient safety and comfort, via open‐face masks,^1^, ^9^, ^10^, ^11^ but also the reproducibility of inter‐fractional patient positioning and treatment interruption if the patient moves.^12^ SGRT is also used for intra‐fraction motion monitoring and respiratory gating techniques.^11^, ^13^ In addition, Manger et al. stated that SGRT for linac‐based radiosurgery could be a surrogate for the position of intracranial lesions.^1^ SGRT and IGRT can provide complementary imaging information during patient positioning and throughout treatment, which may improve target localization.^4^, ^13^

The ExacTrac Dynamic (EXTD) system, version 1.0 (Brainlab AG, Munich, Germany), installed in our institution in June 2020, is a combined SGRT and IGRT system used for patient positioning, monitoring and tumor targeting. The system is able to provide intra‐fractional positioning information of the bony anatomy via oblique stereoscopic X‐ray imaging of the patient in parallel to real‐time 3D surface imaging, including thermal information, for continuous motion detection during treatment delivery.^13^ In contrast to other systems, only one optical camera is used but thermal information creates an additional dimension, which is assumed to improve tracking accuracy.^14^

In RT, especially for SRT or SRS treatments, the congruence between the radiation isocenter and imaging isocenters needs to be verified, for coplanar and noncoplanar treatments.^15^, ^16^, ^17^

Several studies investigating the accuracy of positional correction systems have been conducted over the past years.^4^, ^11^, ^12^, ^15^, ^16^, ^18^, ^19^, ^20^, ^21^, ^22^ Koubuchi et al. performed a study where the accuracy of the positional correction after treatment couch rotation was investigated and an accuracy of 0.5 mm was found for the ExacTrac X‐ray system, version 5.5.2.^22^ Another study comparing the isocenter localization accuracy of the ExacTrac X‐ray and the on‐board CBCT (Varian Medical Systems, Palo Alto, CA) systems was performed, demonstrating that the isocenter agreement was in the range of 1 mm.^4^ Ma et al. analyzed and compared the accuracy achieved by SGRT and CBCT in breast cancer RT for inter‐fractional patient positioning measurements and concluded that the two systems showed good agreement, suggesting that SGRT could be used as an effective measure to increase patient positioning precision during breast cancer RT treatment, complementary to CBCT.^12^, ^23^, ^24^, ^25^ Moreover, Swinnen et al. demonstrated that submillimeter accuracy might be achieved by a linac equipped with an optical surface tracking system for a noncoplanar single isocenter SRS treatment for multiple brain metastases.^15^ Chow et al. investigated the performance of the EXTD system compared to CBCT with a phantom study.^26^

Our study aimed to provide additional information about the geometric accuracy of EXTD and its workflow in a clinical environment. The investigation of the performance of the EXTD system and its IGRT components, such as the spatial drift of the optical/thermal system was investigated. Both optical/thermal‐ and the stereoscopic X‐ray imaging systems were subjected to several tests and the comparison to CBCT was also performed. Moreover, as the system is mainly used in SRT and SRS treatments, further measurements recommended in the literature, like a hidden target Winston–Lutz (WL) and an end‐to‐end IGRT test, were included.^27^, ^28^ Additionally, intra‐fractional X‐ray and combined optical/thermal motion data from a study with 14 patients with intracranial tumors, treated at our institution with open‐face masks, was evaluated.

## 2 METHODS

### 2.1 EXTD system

The EXTD was used in all experiments. It combines the following in‐room SGRT and IGRT strategies:

– Optical structured light scanning (SLS)
– Thermal imaging
– Oblique stereoscopic kilovoltage (kV) X‐ray imaging

The system consists of an optical/thermal imaging device, which contains a blue light projector, two stereoscopic high‐resolution cameras, and an integrated thermal camera.^14^ The optical/thermal imaging device is positioned centrally above the treatment couch. Moreover, two kV X‐ray tubes are mounted in the bunker floor, projecting obliquely onto two ceiling mounted flat panel detectors, with a 300 × 300‐mm^2^ radiation sensitive area.^29^, ^30^ The geometrical radiation field size at the isocenter is 180 × 180 mm^2^.^30^

The structured light projector emits a pattern onto the patient surface detected by the two optical cameras. The camera images of this pattern are used to calculate a 3D map of the patient surface. Moreover, a 2D thermal matrix is created from the patient's heat signal taken by the integrated thermal camera. These two matrices are matched in order to calculate a hybrid 3D+thermal matrix containing spatial and thermal information of each point of the patient surface.^14^

The internal anatomy can be verified through paired stereoscopic kV X‐rays. These images are then compared to the digitally reconstructed radiographs (DRRs), which are calculated from the planning computed tomography (CT) scan and the isocenter position, yielding a rigid body transformation.^31^

All measurements were performed in a clinical environment. The EXTD system was installed at an Elekta Versa HD linac equipped with the HexaPOD evo RT System (Elekta AB, Stockholm, Sweden) controlling the treatment couch movement within 6 degrees of freedom (DoF) with an accuracy of ±0.2 mm.^32^ The linac was equipped with an Agility multileaf collimator consisting of 160 leaves with a leaf width of 5 mm at the isocenter.

### 2.2 EXTD clinical workflow

#### 2.2.1 EXTD monitoring preparation

Once the planning CT and the treatment plan are imported into EXTD, the monitoring strategy needs to be prepared before treatment. This implies the selection of the treatment indication template, the review of some ExacTrac settings, like the surface tracking and X‐ray displacement tolerances, auto X‐ray triggers, the restriction of the CT volume to improve X‐ray to DRR fusion, the adjustment of the patient skin tone setting (light, fair, medium or dark), among others.^30^

#### 2.2.2 Patient positioning

Pre‐positioning is first used to approximately position the patient before the treatment, by matching the live 3D optical surface, without thermal information, to the reference contour extracted from the treatment planning CT. A 3‐DoF translation shift is sent to the treatment table, and the patient is moved.

After the patient is roughly positioned, an area of interest (AOI), as a surrogate of the movement of the planning target volume, needs to be selected. The AOI's purpose is to track the patient's movement using surface tracking, while providing thermal information. Optical and thermal surface information are used during the monitoring mode, where the AOI is treated as a single rigid area.^30^ The quality of the monitoring depends not only on the size of the AOI but also on the topology of the surface.^30^ Moreover, for a reliable tracking of the patient movement, the AOI should not include parts of the linac or any external systems.^30^

For final positioning, a pair of X‐ray images is acquired and registered to the DRRs. A 6‐DoF X‐ray‐based correction shift of the patient is sent to the robotic couch Elekta HexaPOD evo RT System. Another pair of X‐ray images is taken to confirm the patient's position, and the process is iterated until the remaining deviation from the actual position, and the reference position is within the specified tolerances, at which point the patient is considered to be positioned with sufficient accuracy.^31^ After the last pair of X‐ray images is acquired and the patient is correctly positioned, the optical surface reference information is updated.^14^

#### 2.2.3 Patient monitoring

The patient monitoring mode is used to track intra‐fractional patient motion. The 3D live optical surface information is projected over the thermal image plane and 3D surface points and 2D thermal data are correlated, using the Perspective‐n‐Point algorithm.^14^ The 3D optical/thermal information is constantly compared to the reference image, X‐ray images are triggered according to predefined settings and if the surface tracking exceeds the predefined tolerances. Every time stereoscopic X‐ray images are acquired, and if patient positioning is within the tolerance, a new optical/thermal imaging reference is established.^30^

### 2.3 Description of the experiments

**FIGURE 1:**
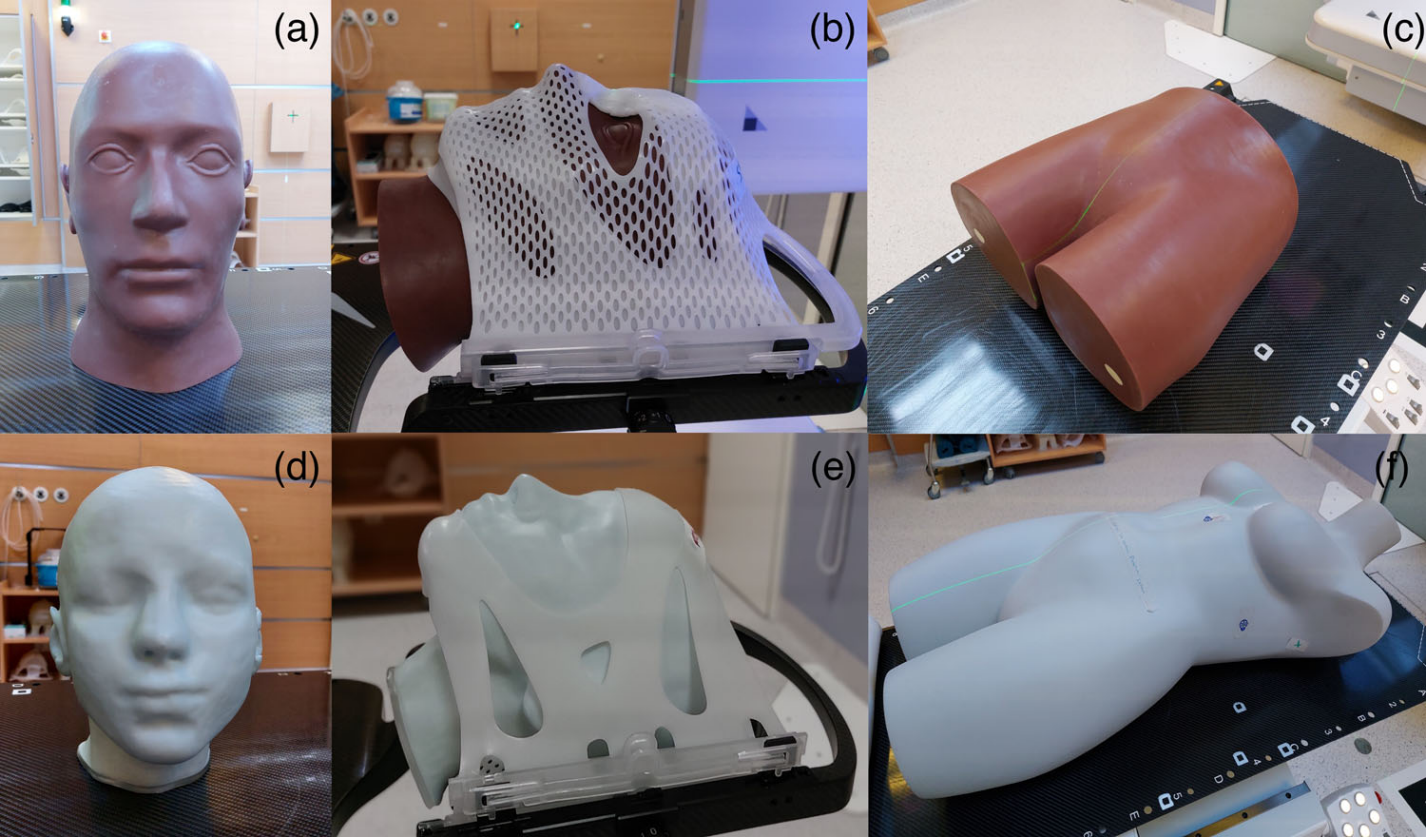
Anthropomorphic phantoms: (a) cranial verification phantom, (b) cranial phantom immobilized on the treatment couch with a cranial 4Pi stereotactic mask, (c) pelvic verification phantom, (d) 3D‐printed head phantom with bone equivalent material, (e) head phantom immobilized on the treatment couch with a cranial 4Pi open face mask, and (f) abdominothoracic phantom with bone‐equivalent material and a distinct heat signature

For the EXTD system, when preparing the monitoring strategy, the skin tone was adjusted for every phantom individually. For both cranial and pelvic verification phantoms, Figure 1a,c, respectively, the chosen skin tone was “dark,” whereas for the 3D‐printed head phantom and the abdominothoracic phantom, Figure 1d,f, respectively, the skin tone was adjusted to “light.” All experiments were performed in a treatment room, with air temperature at ∼21°C.

#### 2.3.1 EXTD and CBCT: positioning consistency

The differences between EXTD and CBCT image registrations, and therefore the corresponding positioning consistency of both systems, were measured using two anthropomorphic phantoms of two different anatomical regions: a cranial verification phantom (Brainlab AG, Munich, Germany) and a pelvic verification phantom (Brainlab AG, Munich, Germany) (Figure 1a–c). The phantoms were scanned with a CT scanner (Toshiba Aquilion LB, Canon Medical Systems, Japan), with a slice thickness of 3 mm for the pelvic phantom (0.7 × 0.7 × 3 mm^3^), and 1 mm for the head phantom (0.9 × 0.9 × 1 mm^3^) according to the recommendations for linac SRS/SBRT quality assurance programs.^33^, ^34^, ^35^, ^36^ For each phantom, six arbitrary isocenters were defined manually and the CT datasets were transferred to both EXTD and the CBCT system XVI version 5.0.4 (Elekta AB, Stockholm, Sweden).

##### Setup procedure and positioning consistency

Before starting the measurements, the radiation isocenter and the imaging isocenters of the EXTD and CBCT systems were aligned to the same reference point, using the cranial phantom with an inserted ball bearing (BB), which was fixed to the treatment couch with a cranial 4Pi stereotactic immobilization system (Brainlab AG, Munich, Germany) (Figure 1b).

**FIGURE 2:**
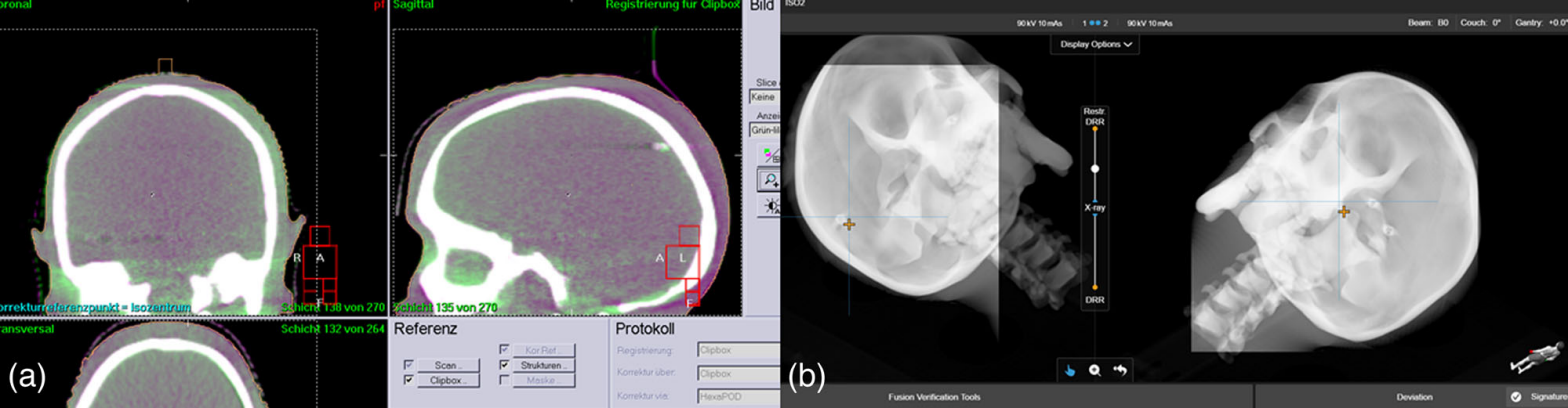
Registration of actual positioning image with planned positioning image, of the cranial verification phantom, by both systems: (a) cone‐beam computed tomography (CBCT) and (b) stereoscopic X‐ray imaging

To compare the positioning accuracy of EXTD and CBCT imaging, both the cranial phantom along with the cranial stereotactic mask and the pelvic phantom were used. The phantoms were pre‐positioned using EXTD surface data. Afterward, EXTD stereoscopic X‐ray images and a CBCT scan were acquired and the calculated correction shifts in 6 DoF of both systems were compared. Finally, the shifts calculated by the EXTD system were applied by moving the treatment couch with the HexaPOD evo RT system. Another pair of stereoscopic X‐ray images and a second CBCT scan were acquired to determine the residual shift after positioning correction. In Figure 2a,b, the fusion of current and planned positioning images by both systems, CBCT and EXTD X‐ray imaging, is shown.

These two positioning methods resulted in correction shifts which were compared according to the following equation:

(1) $$\Delta d_{\mathrm{CBCT}-\mathrm{Xray}}=d_{\mathrm{CBCT}}-d_{\mathrm{Xray}}$$

for the lateral (Δ*d*_CBCT‐Xray,_ *_X_*), longitudinal (Δ*d*_CBCT‐Xray,_ *_Y_*), and vertical (Δ*d*_CBCT‐Xray,_ *_Z_*) translational directions and pitch (Δ*d*_CBCT‐Xray,PITCH_), roll (Δ*d*_CBCT‐Xray,ROLL_), and yaw (Δ*d*_CBCT‐Xray,YAW_) rotational DoF, according to the patient coordinate system.

To investigate the stability and reproducibility of the measurements, this experiment was conducted for each of the six isocenters in two separate time points within ∼10 months difference. The second measurement consisted in repeating the procedure five times.

Imaging settings for CBCT and EXTD used for the measurements are indicated in Table 1. Rigid registration and automatic fusion, based on bony features, were performed by the proprietary matching algorithms of both software systems.

**TABLE 1:** Cone‐beam computed tomography (CBCT) and ExacTrac Dynamic (EXTD) X‐ray exposure parameters

		Head phantom	Pelvic phantom
**CBCT**	Tube voltage (kV)	100	120
	Total mAs	18.3	264
	Gantry angle rotation span	200°	360°
	kV Collimator/kV filter axial FOV	S20/F0 27 cm (small)	M20/F1 41 cm (medium)
**ExacTrac Dynamic**	Tube voltage (kV)	90	120
	mAs	10	20

#### 2.3.2 EXTD: optical/thermal‐, stereoscopic X‐ray imaging, and radiation isocenter

**FIGURE 3:**
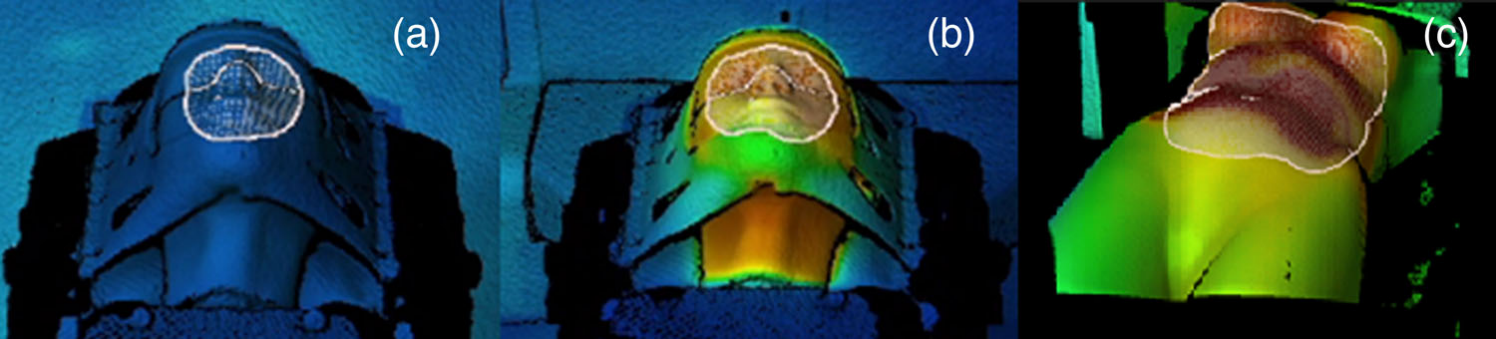
3D surface image of phantoms at different temperatures, projected over a thermal image plane: (a) head phantom with a cold surface, (b) head phantom with a warm surface, and (c) abdominothoracic phantom with a warm surface

Two dedicated anthropomorphic phantoms with a distinct thermal signature were used for this part of the study (Figure 1d–f):

- A head phantom, 3D‐printed from bone‐equivalent materials with three spherical 5‐mm‐diameter‐embedded BBs (RTsafe, Greece; Figure 3a,b, respectively), was filled with water. The phantom was immobilized to the couch using a cranial 4Pi open‐face mask (Brainlab AG, Germany) and scanned with a CT scanner with a slice thickness of 0.5 mm.^33^, ^34^, ^35^, ^36^ The central BB was chosen as the isocenter (BB0) and delineated on the planning CT dataset (0.6 × 0.6 × 0.5 mm^3^) using the clinical treatment planning system Oncentra MasterPlan Version 4.5 (Elekta AB, Stockholm, Sweden).
- An abdominothoracic phantom with bone‐equivalent materials and a distinct and constant heat signature (Brainlab AG, Germany) was scanned with a CT scanner, with a slice thickness of 3 mm (1 × 1 × 3 mm^3^). It was positioned on the treatment couch and no immobilization device was used. One isocenter was chosen, located in the spinal column in the thoracic region.

The CT images of the phantoms and corresponding treatment plans were transferred to EXTD and were used as reference images for the image registration.

##### 2.3.2.1 Spatial drift

Following the recommendations of TG‐147 as well as the TG‐302 reports,^27^, ^37^ measurements to assess the spatial drift of the optical/thermal system were conducted. They were performed with the abdominothoracic phantom (Figure 1f) with cold and warm surfaces (Figure 3c) in the abdominal region. The 3D camera has two systems subject to a warm‐up phase: the system power and the blue light projector. The system power was on for an extended time (>1 h). However, in order to simulate a clinical situation, that is, when this system is not necessarily used every consecutive patient, the blue light projector was switched off for 30 min. The phantom was placed on the treatment couch at 0° and positioned using the EXTD system. The stability of the system was then evaluated by continuing to sample manually the phantom's position in the lateral, longitudinal, and vertical directions, every 0.5 min for 70 min.

##### 2.3.2.2 Influence of the surface temperature

###### 2.3.2.2.1 Cold and warm surface: stability of the optical/thermal imaging positioning values during the delivery of a volumetric modulated arc therapy (VMAT) plan

The head phantom was filled with water at room temperature (labeled “cold surface”) or with warm water (≈41°C) (labeled “warm surface,” surface temperature within 36–32°C over the course of the measurements) (Figure 3a,b) and was immobilized on the treatment couch at 0° and positioned using the EXTD system. In the monitoring mode, the optical/thermal imaging positioning values were monitored in all translational and rotational dimensions and recorded in a log file during the delivery of a VMAT plan, taking ∼12 min. For the cold and the warm surface, the resulting positioning values, *d*_ST,_ *_X_*, *d*_ST,_ *_Y_*, *d*_ST,_ *_Z_*, *d*_ST,PITCH_, *d*_ST,ROLL_, and *d*_ST,YAW_, in the lateral, longitudinal, vertical directions, as well as for pitch, roll and yaw, respectively, were compared and reported. The Mann–Whitney *U* test was used to investigate differences between a cold and a warm surface.

###### 2.3.2.2.2 Cold and warm surface: couch/phantom displacements, optical/thermal‐ and stereoscopic X‐ray imaging

Further measurements with the head phantom (cold and warm surface) (Figure 3a,b), positioned on the treatment couch at 0°, were conducted. Twenty five couch displacements were performed in two distinct time points 9 months apart and repeated four times in order to investigate the stability and reproducibility of the measurements. The couch displacements were performed in all three directions, lateral, longitudinal, and vertical in the range of 0–3 mm, and the optical/thermal‐ and the stereoscopic X‐ray imaging positioning values were compared.

A similar procedure with the head phantom and noncoplanar couch angles (90°, 45°, 315°, and 270°) was carried out. The phantom's surface was warm to simulate the body temperature. Twenty couch displacements were performed in all three translational directions, in the range of 0–3 mm.

The comparison between the optical/thermal and X‐ray imaging positioning systems was performed for the lateral, longitudinal, and vertical directions, relative to the patient coordinate system, according to the following equation:

(2) $$\Delta d_{\mathrm{HEAD}}=d_{\mathrm{Xray}}-d_{\mathrm{ST}}$$

The Mann–Whitney *U* test was used to investigate differences between a cold and a warm surface, at couch 0°. Additionally, for a warm surface, the deviation of the positioning values for both imaging methods was also analyzed for a treatment couch at 0° when compared to other couch angles.

For the abdominothoracic phantom, similar measurements were executed. The following seven couch angles were chosen: 90°, 60°, 30°, 0°, 330°, 300°, and 270° and to simulate an RT treatment in the torso region, where no immobilization system is usually used, 45 random phantom displacements were performed. The optical/thermal‐ and the stereoscopic X‐ray imaging positioning values were compared for both a cold and a warm surface, according to the following equation:

(3) $$\Delta d_{\mathrm{ABDOMINOTHOR}}=d_{\mathrm{Xray}}-d_{\mathrm{ST}}$$

where Δ*d*_ABDOMINOTHOR,_ *_x_*, Δ*d*_ABDOMINOTHOR,_ *_Y_*, and Δ*d*_ABDOMINOTHOR,_ *_Z_* represent the lateral, longitudinal, and vertical directions, respectively.

##### 2.3.2.3 Warm surface: optical/thermal‐, stereoscopic X‐ray imaging positioning and radiation isocenter

To investigate and compare the accuracy of the optical/thermal‐ and stereoscopic X‐ray imaging positioning (IGRT‐derived isocenter) and their correlation with the radiation isocenter position, the setup errors using MV portal images, optical/thermal‐ and X‐ray imaging positioning were investigated. The head phantom was filled with warm water (≈41°C) and positioned using the EXTD system. The isocenter of the plan was located at the center of BB0, and reference setup square fields 2 × 2 cm^2^ (25 MU) centered in the BB0 were created.

With the treatment couch at 0°, five couch displacements with a maximal amplitude of 2 mm in all three translational directions were applied. After each couch displacement, a WL test was performed, with the gantry at cardinal angles (0°, 90°, 180°, and 270°), used as an indicator of the positional correction accuracy. To provide a more representative sample of all possible couch angles for our SRT or SRS treatments, this test was also performed at noncoplanar couch angles (90°, 45°, 315°, and 270°) and gantry at 0°. For each couch angle, four couch displacements with a maximal amplitude of 2 mm in the lateral and longitudinal directions, *s_X_* and *s_Y_*, respectively, were performed. Both reference position and the couch displacements were analyzed using the Winston–Lutz module of Pylinac, version 2.5^38^, ^39^ for processing the WL‐type EPID images. The optical/thermal‐ and the stereoscopic X‐ray imaging positioning values were both compared to the MV portal image setup errors, according to the following equations:

(4) $$\Delta d_{\mathrm{MV}-\mathrm{IGRT},\mathrm{ST}}=d_{\mathrm{MV}}-d_{\mathrm{ST}}$$

(5) $$\Delta d_{\mathrm{MV}-\mathrm{IGRT},\mathrm{Xray}}=d_{\mathrm{MV}}-d_{\mathrm{Xray}}$$

where Δ*d*_MV‐IGRT,ST,_ *_X_*, Δ*d*_MV‐IGRT,ST,_ *_Y_*, and Δ*d*_MV‐IGRT,ST,_ *_Z_* represent the discrepancies between MV portal imaging and optical/thermal positioning values in the lateral, longitudinal, and vertical directions, respectively. Δ*d*_MV‐IGRT,Xray,_ *_X_*, Δ*d*_MV‐IGRT,Xray,_ *_Y_*, and Δ*d*_MV‐IGRT,Xray,_ *_Z_* represent the discrepancies between MV portal imaging and X‐ray imaging positioning values in the lateral, longitudinal, and vertical directions, respectively.

##### 2.3.2.4 Hidden target test

The hidden target test resembles the traditional workflow of IGRT‐based treatments and is performed to assess the coincidence of both imaging and radiation isocenters. This procedure is executed regularly at our institution, using the anthropomorphic cranial verification phantom, immobilized on the treatment couch with a cranial stereotactic mask (Figure 1a,b), and positioned with EXTD. This phantom has a radiopaque spherical 5‐mm‐diameter‐embedded BB, which was chosen as the isocenter, and it was scanned with a slice thickness of 1 mm (0.6 × 0.6 × 1 mm^3^) according to the recommendations for linac SRS/SBRT quality assurance programs. A treatment plan with square fields of 2 × 2 cm^2^ centered in the isocenter with gantry at 0°, 90°, 180°, and 270° was used. The results of 40 WL tests (performed between March 2021 and March 2022), for which the recommended accuracy is 1 mm for SRS treatments,^27^, ^28^, ^37^ were included in this study. Δ*d*_WL,_ *_X_*, Δ*d*_WL,_ *_Y_*, and Δ*d*_WL,_ *_Z_* represent the residual differences between IGRT‐aligned isocenter and the radiation field isocenter, in the lateral, longitudinal, and vertical directions, respectively.

##### 2.3.2.5 End‐to‐end IGRT test

Following the recommendations of the TG‐147 and TG‐302 reports,^27^, ^37^ an end‐to‐end IGRT test, which aims to assess the entire clinical IGRT process, was also performed. After making a new stereotactic mask, the acquisition of a new CT scan, and the creation of a new treatment plan, the same procedure as the hidden target test was followed. The WL test was executed 12 times (repeated exposures while moving the phantom between exposures).

#### 2.3.3 Patient data

A total of 14 patients with intracranial tumors treated at our institution during the first 3 months of 2021 underwent normofractioned cranial RT treatments. Patients were immobilized to the couch using a cranial open‐face mask (Brainlab AG, Germany and IT‐V, Innsbruck, Austria), positioned and monitored with EXTD, and 142 fractions were analyzed. To detect intra‐fractional motion and quantify the deviation between planned and current position during the treatment, optical/thermal surface information from the area within the face opening was collected and recorded in a log file. Simultaneously, patients were monitored by stereoscopic X‐ray imaging, acquired at gantry positions 0°, 90°, 180°, and 270°, providing anatomical information about the position of bony structures.

In Figure S1, an exemplary patient monitoring with EXTD is shown, with stereoscopic X‐ray and optical/thermal imaging information, as well as a graphical representation of the intra‐fractional motion during the treatment.

The 806 optical/thermal‐ and stereoscopic X‐ray imaging positioning values were recorded. The differences between positioning values provided by both systems were evaluated and compared, according to the following equation:

(6) $$\Delta d_{\mathrm{PAT}}=d_{\mathrm{Xray}}-d_{\mathrm{ST}}$$

where Δ*d*_PAT,_ *_X_*, Δ*d*_PAT,_ *_Y_*, Δ*d*_PAT,_ *_Z_*, Δ*d*_PAT,PITCH_, Δ*d*_PAT,ROLL_, and Δ*d*_PAT,YAW_ represent the differences in the positioning values in the lateral, longitudinal, and vertical directions, as well as for pitch, roll and yaw, respectively.

### 2.4 Statistical analysis

The IBM SPSS Statistics Version 26.0 software (IBM Corporation, Armonk, New York) was used to perform Mann–Whitney *U* tests to investigate differences of the positioning values for optical/thermal imaging between a cold and a warm surface, as well as to analyze the difference between both imaging methods, optical/thermal‐ and stereoscopic X‐ray imaging. Differences were considered statistically significant for *p* value ≤0.05.

## 3 RESULTS

### 3.1 EXTD and CBCT: positioning consistency

The positional differences between EXTD stereoscopic X‐ray imaging and CBCT for the cranial and the pelvic phantoms, as calculated with Equation (1), are shown in Table 2. For the cranial phantom, the largest translational deviation observed was in the lateral direction, showing a median difference of 0.4 mm. For the pelvic phantom, the largest translational deviation was also observed in the lateral direction, with a median of 0.3 mm. Nevertheless, all differences were within the submillimeter range. Regarding the rotational DoF, deviations were also similar and the largest median difference was 0.4°.

**TABLE 2:** The difference between the stereoscopic X‐ray and cone‐beam computed tomography (CBCT) positional shifts measured using two anthropomorphic phantoms, each phantom with six different isocenter locations (median and IQR)

Stereoscopic X‐ray imaging vs. CBCT							
		Δ*d*_CBCT‐Xray,_ *_X_* (mm)	Δ*d*_CBCT‐Xray,_ *_Y_* (mm)	Δ*d*_CBCT‐Xray,_ *_Z_* (mm)	Δ*d*_CBCT‐Xray,PITCH_ (°)	Δ*d*_CBCT‐Xray,ROLL_ (°)	Δ*d*_CBCT‐Xray,YAW_ (°)
**Cranial verification phantom**	Initial correction	0.4 [0.1; 0.9]	−0.1 [−0.2; 0.1]	0 [−0.3; 0.4]	0.4 [0; 1.1]	−0.1 [−0.6; −0.1]	−0.3 [−0.4; −0.2]
	After correction	0.4 [0.3; 0.4]	0 [−0.1; 0.1]	−0.2 [−0.4; 0.1]	0 [−0.1; 0.1]	0 [−0.1; 0.1]	0 [0; 0.1]
**Pelvic verification phantom**	Initial correction	0.3 [0.2; 0.4]	−0.1 [−0.6; 0.1]	0.1 [−0.3; 0.6]	0.1 [0.1; 0.3]	0.2 [0.1; 0.5]	−0.2 [−0.2; −0.1]
	After correction	0.2 [0.1; 0.3]	0.1 [−0.2; 0.5]	0.1 [−0.2; 0.3]	0.1 [0; 0.1]	−0.1 [−0.3; 0.2]	0 [−0.1; 0]

Repetition of the measurements (five times) after 10 months yielded similar results (Table S1).

### 3.2 EXTD: optical/thermal‐ and stereoscopic X‐ray imaging and MV beam

#### 3.2.1 Spatial drift

**FIGURE 4:**
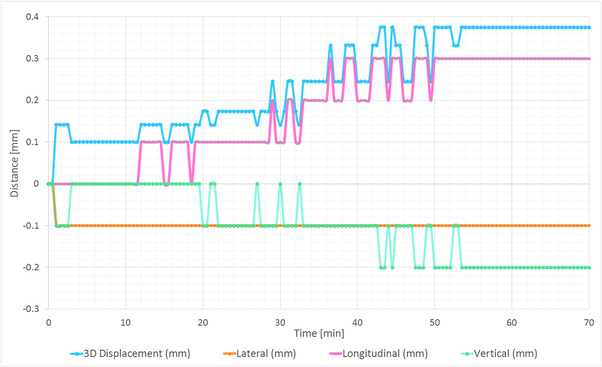
Surface‐derived position deviations recorded from the initial position in the lateral, longitudinal, and vertical directions, as well as the 3D displacement vector of the total deviation, for the abdominal phantom with a warm surface

Measurements using the abdominothoracic phantom with a warm surface showed drifts up to 0.4 mm. In Figure 4, the deviations in all three translational directions are plotted, as well as the vector length of the deviation, where the maximal deviation is observed in the longitudinal direction, 0.3 mm. The rotational drifts did not exceed 0.1° in all three directions.

For a cold surface, the drifts reached 0.8 mm. The largest deviations were also seen in the longitudinal direction, 0.7 mm, and the rotational drifts were below 0.3° in all three directions. The deviations in all three translational directions are plotted in Figure S2.

#### 3.2.2 Influence of the surface temperature

##### Cold and warm surface: stability of the optical/thermal imaging positioning values during the delivery of a VMAT plan

The variance of the optical/thermal imaging positioning values (*d*_ST_) for a cold and a warm surface, during the delivery of a VMAT plan (∼12 min), was analyzed (Table S2). The recorded values for both temperatures were very similar, and the difference was always <0.07 mm and <0.05°. A Mann–Whitney *U* test was performed to investigate whether the differences reported by the surface optical/thermal imaging positioning system for a cold and a warm surface were statistically significant. The test showed that there was no significant difference in *d*_ST,Y_ and *d*_ST,ROLL_ when comparing a cold and a warm surface (*p* > 0.05), as opposed to the other DoF, which presented *p* ≤ 1 × 10^−5^. However, the median of the deviations observed was always below 0.08 mm and 0.05°. The optical/thermal imaging positioning values recorded in the log file present 0.01‐mm precision.

##### Cold and warm surface: couch/phantom displacements, optical/thermal‐ and stereoscopic X‐ray imaging

**FIGURE 5:**
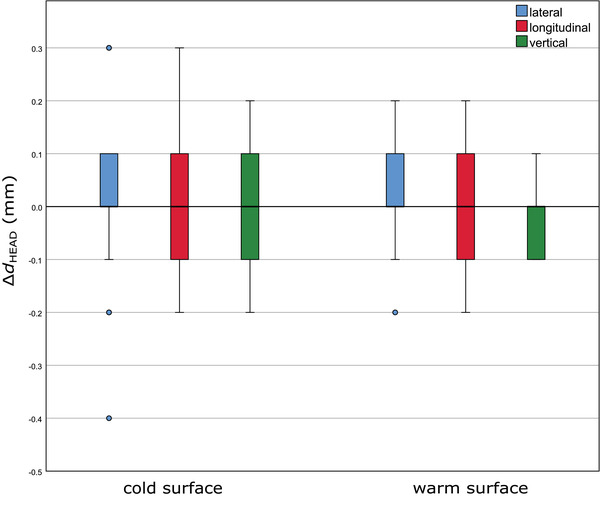
Boxplots of the distribution of the differences between optical/thermal‐ and X‐ray imaging positioning values (25 displacements at couch 0°) for a cold and a warm surface of the head phantom. The results for the different directions, lateral, longitudinal, and vertical, are plotted in blue, red, and green, respectively. The boxplots indicate the spread of the central 50% of the data, denominated as IQR. The median, the 25th (Q_1_) and the 75th (Q_3_) percentiles are also shown. The upper and the lower whiskers represent data outside the IQR but inside the range defined by 1.5 × IQR. Outliers are defined as values outside the whiskers’ range.

For the head phantom with a cold and a warm surface, treatment couch at 0°, boxplots of the distribution of the differences between optical/thermal‐ and X‐ray imaging positioning values for 25 couch displacements are presented in Figure 5. For a warm surface, the differences between both imaging positioning modalities were slightly less scattered than the ones for a cold surface. However, no median differences in any direction were observed. The maximum deviation observed for a cold surface was 0.4 mm in the lateral direction, whereas for a warm surface the maximal deviation was 0.2 mm, in both lateral and longitudinal directions. In addition, the Mann–Whitney *U* test showed no significant difference between a cold and a warm surface regarding the distribution of the differences between optical/thermal‐ and X‐ray imaging positioning values (*p* > 0.05).

The same 25 couch displacements were performed 9 months later (four repetitions) and similar results were found (Figure S3).

Moreover, similar measurements (20 couch displacements) were performed with the head phantom for the noncoplanar couch angles 90°, 45°, 315°, and 270°, in the lateral, longitudinal, and vertical directions. Boxplots of the distribution of the differences between optical/thermal‐ and X‐ray imaging positioning values for the head phantom (Equation 2) are presented in Figure S4. The largest deviations were observed for couch angles 45° and 315°, and the maximal deviation recorded was 0.4 mm in the lateral direction. The median differences were 0 mm for all couch angles, except for couch 90°, in the lateral direction, where this value reached 0.1 mm. The smallest deviations were observed for couch angles 0° and 270°, in all three directions, with a maximal deviation of 0.2 mm. The result of the Mann–Whitney *U* test between the couch at 0° and the other four noncoplanar couch angles were statistically nonsignificant, *p* > 0.05.

**FIGURE 6:**
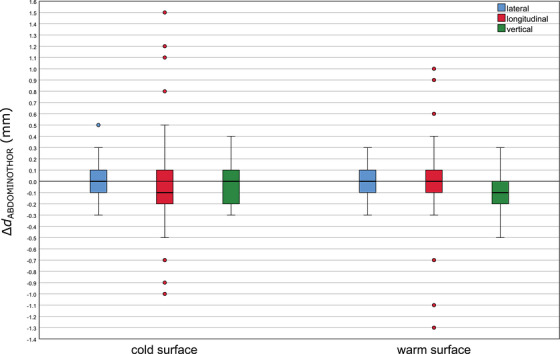
Boxplots of the distribution of the differences between optical/thermal‐ and X‐ray imaging positioning values (45 measurements at 7 couch angles) for a cold and a warm surface of the abdominothoracic phantom. The results for the different directions, lateral, longitudinal, and vertical are plotted in blue, red, and green, respectively. The boxplots indicate the spread of the central 50% of the data, denominated as IQR. The median, the 25th, and the 75th percentiles are also shown. The upper and the lower whiskers represent data outside the IQR but inside the range defined by 1.5 × IQR. Outliers are defined as values outside the whiskers’ range.

For the abdominothoracic phantom, to simulate an RT treatment in the torso region, where no immobilization system is usually used, 45 random phantom displacements, with displacements between 4.6 and 65.6 mm, were performed for seven different couch angles. The deviations observed between the optical/thermal‐ and the stereoscopic X‐ray imaging positioning values, with a cold and a warm surface, are shown in Figure 6. The median differences, for both cold and warm surface, were very similar and below 0.2 mm. The largest deviations were observed in the longitudinal direction, with a maximal deviation of 1.5 mm for a cold surface and 1.3 mm for a warm surface. The Mann–Whitney *U* test showed no significant difference between both temperature surfaces regarding the distribution of the differences between optical/thermal‐ and X‐ray imaging positioning values (*p* > 0.05).

#### 3.2.3 Warm surface: optical/thermal‐, stereoscopic X‐ray imaging positioning and radiation isocenter

##### *Couch 0*°

**FIGURE 7:**
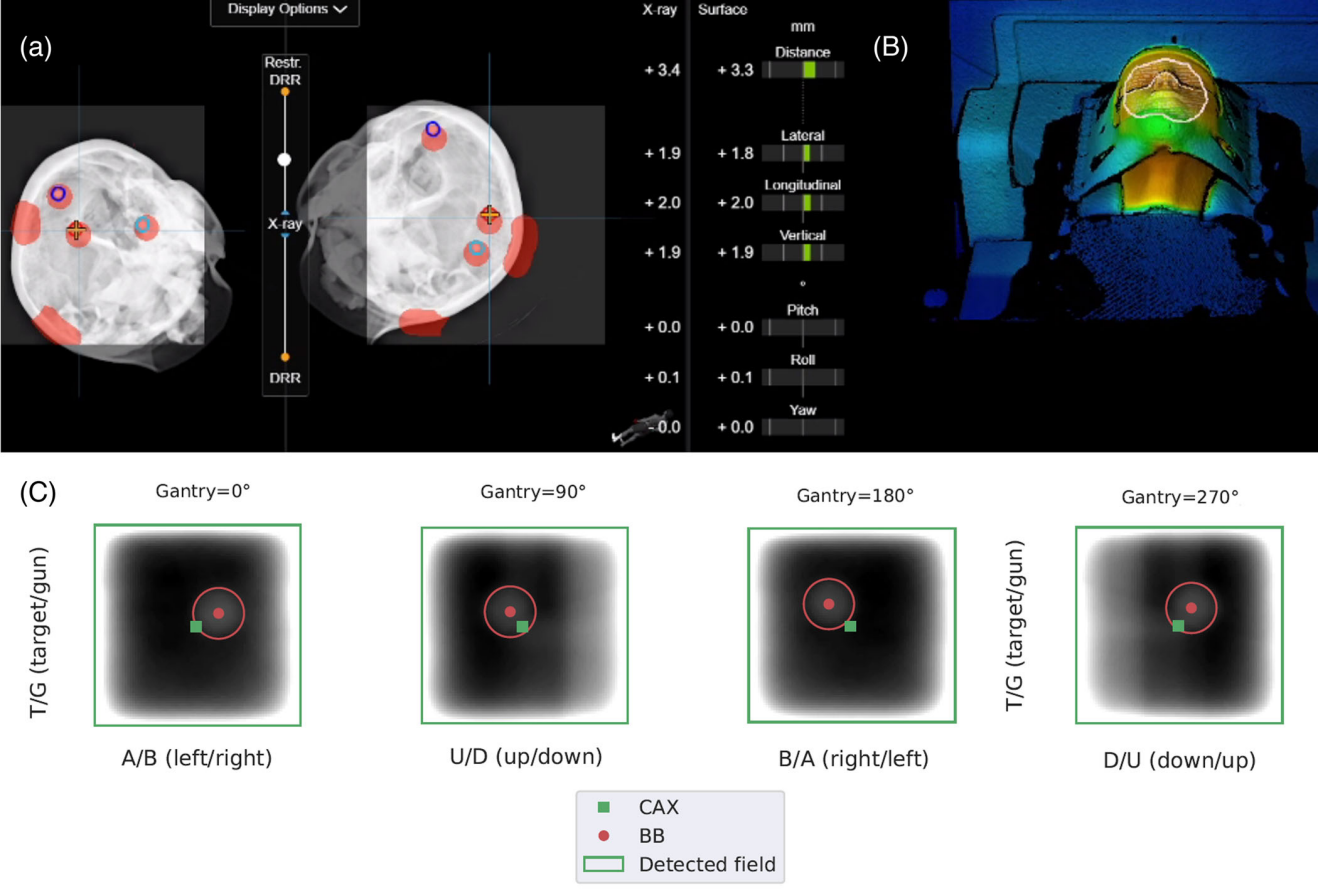
Different methods of monitoring the phantom's position: (a) stereoscopic X‐ray imaging and (b) optical/thermal imaging. The values in the center indicate the distance from the planned and the current position of the phantom provided by both systems; (c) EPID images processed with Pylinac, indicating the irradiated 2 × 2‐cm^2^ MV beam from all four gantry angles, the MV beam center (CAX), and the position of the detected BB

The deviations between the positioning values, reported by optical/thermal‐ and stereoscopic X‐ray imaging, respectively, and the displacement of the BB0 center from the MV beam center (Equations 4 and 5), after five couch displacements in all three directions of a maximal amplitude of 2 mm, are shown in Table 3. The median differences for both imaging positioning modalities in comparison to MV portal imaging positioning were not larger than 0.3 mm. The deviations between optical/thermal‐ and MV portal imaging differ by at maximum 0.1 mm from the corresponding deviation between stereoscopic X‐ray‐ and MV portal imaging. Figure 7 shows the different methods of monitoring the head phantom's position, with stereoscopic X‐ray imaging and optical/thermal imaging.

**TABLE 3:** The difference (median and IQR) between the positioning values, reported by optical/thermal‐ and stereoscopic X‐ray imaging, respectively, and the distance between the MV radiation center and the BB's center, in a 2 × 2‐cm^2^ field (25 MU, 6‐MV photons), for five couch translations in all three directions

	Surface/Thermal imaging vs. MV portal image (mm)				Stereoscopic X‐ray imaging vs. MV portal image (mm)			
Gantry angle	Δ*d*_MV‐IGRT,ST,_ *_X_*	Δ*d*_MV‐IGRT,ST,_ *_Y_*	Δ*d*_MV‐IGRT,ST,_ *_Z_*	3D displacement	Δ*d*_MV‐IGRT,Xray,_ *_X_*	Δ*d*_MV‐IGRT,Xray,_ *_Y_*	Δ*d*_MV‐IGRT,Xray,_ *_Z_*	3D displacement
0°	0.2 [0.1; 0.2]	−0.1 [−0.2; −0.1]	–	0.1 [0; 0.1]	0.1 [0; 0.3]	−0.1 [−0.1; 0]	–	0.2 [0.1; 0.2]
90°	–	0 [−0.1; 0]	0.2 [0.2; 0.2]	0.2 [0.2; 0.4]	–	0 [0; 0]	0.2 [0.2; 0.3]	0.3 [0.2; 0.4]
180°	0 [−0.1; 0.1]	−0.1 [−0.1; −0.1]	–	0 [−0.1; 0.1]	−0.1 [−0.1; 0]	0 [−0.1; 0]	–	0.1 [0; 0.1]
270°	–	−0.1 [−0.3; −0.1]	0.3 [0.2; 0.3]	0.1 [0; 0.2]	–	−0.1 [−0.2; 0.1]	0.2 [0.2; 0.3]	0.1 [0.1; 0.2]

*Note*: For each gantry angle, only two of three directions are shown as the information reported by MV portal image positioning is in 2D.

##### Couch rotation

The four applied couch displacements, in the lateral (*s_X_*) and longitudinal (*s_Y_*) directions, and the differences between the positioning values, reported by optical/thermal‐ and stereoscopic X‐ray imaging, respectively, and the displacement of the BB center from the MV beam center (Equations 4 and 5), are shown in Table S3. The maximal displacement observed was for couch angle 315°, for both imaging modalities, compared to MV portal imaging, in the lateral direction: Δ*d*_MV‐IGRT,ST,_ *_X_* reached 0.7 mm, whereas Δ*d*_MV‐IGRT,Xray,_ *_X_* was 0.6 mm. Nevertheless, the 3D displacement was always below 1‐mm difference for all the measurements.

#### 3.2.4 Hidden target test

The median differences between the BB center and the MV beam center, as well as the IQR for all 40 measurements, are shown in Table 4. The median differences in all directions did not exceed −0.7 mm, observed in the longitudinal direction for a gantry angle 180°. However, considering the same direction, a difference of 0.4 mm was found for gantry angle 0. In the lateral direction, the median difference was 0 mm, whereas in the vertical direction, the median did not exceed 0.2 mm. The magnitude of the range of the differences was not higher than 0.4 mm.

**TABLE 4:** Residual difference (median and IQR) between image‐guided radiotherapy (IGRT)‐aligned isocenter (BB's center) and MV radiation field center (median and IQR), in a 2 × 2‐cm^2^ field (25 MU, 6‐MV photons)

Gantry angle								
	0°		90°		180°		270°	
	Δ*d*_WL,_ *_X_* (mm)	Δ*d*_WL,_ *_Y_* (mm)	Δ*d*_WL,_ *_Z_* (mm)	Δ*d*_WL,_ *_Y_* (mm)	Δ*d*_WL,_ *_X_* (mm)	Δ*d*_WL,_ *_Y_* (mm)	Δ*d*_WL,_ *_Z_* (mm)	Δ*d*_WL,_ *_Y_* (mm)
Median	0	0.4	0.2	0.2	0	−0.7	0	0.2
IQR	[−0.2; 0.2]	[0.2; 0.5]	[0; 0.2]	[0; 0.2]	[0; 0.2]	[−0.7; −0.5]	[−0.1; 0.2]	[0; 0.2]

*Note*: Results representing 40 IGRT‐positioned phantom exposures, acquired throughout 1 year.

#### 3.2.5 End‐to‐end IGRT test

The median differences and the IQR range are shown in Table S4. The resulting values are in submillimeter range and agreed with the ones shown in Table 4.

### 3.3 Patient data

The differences between optical/thermal‐ and stereoscopic X‐ray imaging positioning values are shown in Table 5. The mean of the differences was close to 0 in all directions and the standard deviation always below 0.5 mm for translations and below 0.5° for rotations.

**TABLE 5:** Difference between the positioning values reported by optical/thermal‐ and stereoscopic X‐ray imaging monitoring: Mean values for all translations and rotations over 806 recorded values, with the corresponding standard deviations, standard errors of mean, and 95% confidence intervals

	Mean	Standard deviation	Standard errors of mean	95% Confidence interval
Δ*d*_PAT,_ *_X_* (mm)	0.02	0.35	0.01	[−0.01; 0.04]
Δ*d*_PAT,_ *_Y_* (mm)	0	0.35	0.01	[−0.02; 0.02]
Δ*d*_PAT,_ *_Z_* (mm)	0	0.40	0.01	[−0.03; 0.03]
Δ*d*_PAT,PITCH_ (°)	0	0.10	0	[0; 0.01]
Δ*d*_PAT,ROLL_ (°)	0	0.08	0	[−0.01; 0]
Δ*d*_PAT,YAW_ (°)	0	0.09	0	[0; 0.01]

## 4 DISCUSSION

The deviations between CBCT and EXTD positioning systems were found to be in the submillimeter range. Furthermore, both systems proved to be stable over time as experiments repeated 10 months later showed similar results with differences below 1 mm. Due to the regular and independent recalibration of the IGRT isocenters to the radiation isocenter and the inherent uncertainty, they could not be exactly the same. As SRS treatments are performed at this machine, the tolerated deviation between imaging and radiation isocenters is less than 1 mm and therefore the maximal difference between both imaging isocenters could be up to 2 mm. Li et al.^5^ performed a very similar study using a head phantom and 10 isocenter locations, where the absolute differences in the calculated couch residual errors between ExacTrac X‐ray imaging registration and TrueBeam CBCT (Varian Medical Systems, Palo Alto, CA) imaging registration in translational and rotational directions were analyzed, and average residual error differences were found to be <0.5 mm and <0.4°, which agrees with our results. Zollner et al. also investigated the discrepancies between ExacTrac X‐ray and CBCT (Elekta AB, Stockholm, Sweden) imaging positioning for a head phantom, as well as for patients.^40^ They reported median differences below 0.5 mm for all translations, which is similar to our findings. Kim et al. compared the positional accuracy of ExacTrac and on‐board CBCT (Varian Medical Systems, Palo Alto, CA) using a pelvic phantom.^4^ The differences for the translational directions were slightly larger compared to our results, nonetheless comparable and in the range of 1 mm. A similar study with both cranial and pelvis phantoms was performed by Chow et al. to investigate the positional differences detected by EXTD X‐ray and CBCT (Varian) imaging, and comparable results were reported (deviations <0.6 mm and <0.6°).^26^

To assess the influence of the thermal imaging, several tests with phantoms at different temperatures were performed. Spatial drift was investigated over 70 min, for both warm and cold surfaces, after disabling the blue projector light for 30 min. The deviations were higher for a cold surface, reaching ∼0.8 mm, as opposed to 0.4 mm for a warm surface, which suggests a benefit when a patient‐like temperature is present. The largest deviations were observed in the longitudinal direction for both cases. Possible reasons for this finding may be the cylindrically symmetric surface of the abdominal region^26^, ^41^ or the lower resolution of the CT scan of this phantom in the longitudinal. However, more measurements are necessary to determine if there is a permanent tendency for larger discrepancies in the longitudinal direction. Lehmann et al. conducted a similar study on system drift for the C‐Rad Catalyst HD system and reported drifts with a magnitude between 0.7 and 1 mm, comparable to our results for a cold surface.^42^

The investigation of the stability of the optical/thermal positioning values during the delivery of a VMAT plan on a cold and a warm head phantom showed very similar values in all DoFs for both temperature levels. The statistical test indicated significant differences for the lateral and vertical translations as well as for pitch and yaw, between a cold and a warm phantom. The discrepancy was however below 0.08 mm and 0.05°. It should, moreover, be noted that when patients with intracranial tumors receive RT treatment, stereoscopic X‐rays are performed every 90° gantry rotation, and consequently a new optical/thermal imaging reference is taken. In this case, an eventual surface temperature change would not influence the optical/thermal values because they are being constantly recalibrated. As in our experiment optical/thermal positioning values were recorded continuously during 12 min, the reference was thus never updated.

The correlation between optical/thermal‐ and stereoscopic X‐ray imaging positioning values was also investigated. For the head phantom (cold and warm surface), a similar performance was observed with maximal deviations of 0.4 mm and no significant difference between both temperature levels regarding the distribution of the differences between optical/thermal‐ and X‐ray imaging positioning values (*p* > 0.05). Repeated measurements produced similar results. When investigating different couch angles, the maximal deviation was also 0.4 mm, and no significant difference between positioning values for couch 0° and noncoplanar couch angles was found (*p* > 0.05). For the abdominothoracic phantom, the median differences for both surface temperatures were very similar and below 0.2 mm. The largest deviations were observed in the longitudinal direction. However, no significant difference between a cold and a warm surface was found (*p* > 0.05). All these results led to the conclusion that there is a good agreement between optical/thermal‐ and X‐ray imaging. As it was not possible to switch off the thermal camera while performing the measurements with a cold surface, for the cold surface thermal information was also considered. This could be one of the reasons why no significant differences were found between a cold and a warm surface (*p* > 0.05). Another investigation to determine the effectiveness of optical/thermal imaging as an image guidance tool was carried out, claiming that the thermal camera was able to detect surface deviations when a warm surface was present.^43^

The geometric congruence between the IGRT‐derived isocenter and the radiation isocenter, for coplanar and noncoplanar treatments, was also tested, using the head phantom with a warm surface, simulating an SRS treatment. For all couch angles, the differences for both imaging positioning modalities in comparison to MV portal imaging positioning were comparable, always within the submillimeter range. Arp and Carl performed a similar study where the deviation between the linac radiation isocenter and the ExacTrac X‐ray isocenter was investigated.^44^ The reported deviations were slightly higher, in the range from 0.31 to 1.07 mm, but still in good agreement with ours. Huang et al. performed several tests to estimate the targeting accuracy when using image guidance with ExacTrac X‐ray for coplanar and noncoplanar couch angles, with results also consistent with ours, with an overall deviation of 0.5 ± 0.1 mm.^45^

A good agreement between the IGRT‐aligned isocenter and the radiation isocenter was found while analyzing the hidden target tests, as well as the end‐to‐end IGRT test, as the differences between both isocenters did not exceed 0.7 mm. The largest discrepancy was found in the longitudinal direction for gantry angle 180°, with a median difference of −0.7 mm, in contrast to gantry 0°, for which a discrepancy of 0.4 mm was observed. This difference can be attributed to the gantry sag of this specific linac, which is known to be ∼1 mm.

It is important to mention that regarding the skin tone, an adjustment for every patient/phantom individually is possible. This setting is used as an additional information for surface reconstruction. In our experiments, several phantoms with different surface tones were used, and the settings were adjusted accordingly. During our experiments, however, no difference between skin tones was detected in the matter of surface reconstruction, although we did not aim to evaluate this aspect.

The patient study took intra‐fraction motion into account and compared the position of the patient during treatment with the planned position, with both optical/thermal‐ and stereoscopic X‐ray imaging information. It aimed to investigate and compare the constant monitoring capability of the SGRT system with the stereoscopic X‐ray imaging. Values from both systems are in good agreement, as the difference between stereoscopic X‐ray and optical/thermal imaging was very close to 0 in all translational directions and below 0.5° for rotations.

This study investigated the geometric accuracy of EXTD and its IGRT components under different initial setup conditions, using phantoms representing different anatomical regions with different amounts of visible bony anatomy information on the X‐ray images. The magnitude of the displacements between all systems, reported in this study, was within the submillimeter range. This suggests that the image guidance provided by EXTD is accurate at any couch angle, and therefore relevant dosimetric differences in surrounding critical structures and target coverage during the treatment of a patient are not expected.

Additionally, the patient study demonstrated good agreement between the monitoring values of both imaging systems, already demonstrated by our phantom measurements. These findings suggest that the optical/thermal positioning system can be an efficient tool for detecting intra‐fractional motion during therapy.

However, only rigid anthropomorphic phantoms with a fixed relationship between the surface as a surrogate for tumor position and the isocenter were used. This can be considered a limitation of this study, especially in the thoracic and abdominal region, where surface deformation, due to patient organ motion, is clinically observable. Furthermore, results from a larger patient cohort as well as an extension to other treatment sites should also be considered in future investigations.

## 5 CONCLUSIONS

In our institution, CBCT represents the benchmark for patient positioning. EXTD showed to be in close agreement within 0.4 mm with CBCT and is therefore considered a legitimate alternative in specific indications. This is mostly true in cases where positioning relies only on bony structures, and for which patient setup can be accelerated. Especially for treatments with noncoplanar angles, where no CBCT acquisition is possible, the EXTD system presents an advantage. The optical/thermal‐ and stereoscopic X‐ray imaging were found to be in agreement with a maximal deviation of 0.4 mm. When comparing optical/thermal‐ and stereoscopic X‐ray imaging with MV portal imaging positioning, the differences were not larger than 0.7 and 0.6 mm respectively. This study showed that EXTD with its new optical/thermal imaging system is an efficient tool for positioning and monitoring during RT.

## CONFLICT OF INTEREST

The Department of Radiation Oncology of the University Hospital of LMU Munich has research agreements with Elekta, Inc. and Brainlab AG. SC, MN, and PF received speaker honoraria/travel support by Brainlab. PF is currently employed by Brainlab AG. However, at the time of the conception of the article and the analysis of the results, PF was not employed by Brainlab AG. PF declares that his employment had no influence on the study design, the collection, analysis or interpretation of data, on the writing of the manuscript or the decision to submit the manuscript for publication.

## AUTHOR CONTRIBUTIONS

Vanessa Da Silva Mendes and Philipp Freislederer performed the measurements and analysis of data, performed the statistical analysis, and drafted the manuscript. Lili Huang and Katrin Straub assisted in the analysis, reviewed the manuscript, and helped to finalize it. Maximilian Niyazi, Stefanie Corradini, and Daniel Reitz assisted in clinical data collection, reviewed the manuscript, and helped to finalize it. Michael Reiner, Guillaume Landry, Christopher Kurz, and Philipp Freislederer helped design the study, supervised the analysis, reviewed the manuscript, and helped finalize it. Claus Belka, Maximilian Niyazi, and Stefanie Corradini conceived the study, supervised the analysis, reviewed the manuscript, and helped to finalize it. All authors read and approved the final manuscript.

## Supporting information

FigureS1

FigureS2

FigureS3

FigureS4

Figure S1 Patient monitoring. Top left: stereoscopic X‐ray imaging providing information about the internal bony structures. Top right: optical/thermal imaging of the AOI. In the center, each imaging system indicates the difference between the actual and the planned position in all 6 DoF. Bottom: Graphical representation of the intra‐fractional motion during the treatment

Figure S2 Deviations recorded from the initial position in the lateral, longitudinal, and vertical directions, as well as the 3D displacement vector of the total deviation, for a cold surface

Figure S3 Boxplots of the distribution of the differences between optical/thermal‐ and X‐ray imaging positioning values (25 displacements at couch 0°, 4 repetitions) for a cold and a warm surface of the head phantom. The results for the different directions, lateral, longitudinal, and vertical, are plotted in blue, red, and green, respectively. The boxplots indicate the spread of the central 50% of the data, denominated as IQR. The median, the 25th (Q_1_) and the 75th (Q_3_) percentiles are also shown. The upper and the lower whiskers represent data outside the IQR but inside the range defined by 1.5 × IQR. Outliers are defined as values outside the whiskers’ range.

Figure S4 Boxplots of the distribution of the differences between surface/thermal‐ and X‐ray imaging positioning values (20 measurements) for a warm surface, for different couch angles (90°, 45°, 0°, 315°, and 270°). The results for the different directions, lateral, longitudinal, and vertical are plotted in blue, red, and green, respectively. The boxplots indicate the spread of the central 50% of the data, denominated as IQR. The median, the 25th and the 75th percentiles are also shown. The upper and the lower whiskers represent data outside the IQR but inside the range defined by 1.5 × IQR. Outliers are defined as values outside the whiskers’ range.

Table S1 The difference between the stereoscopic X‐ray and CBCT positional shifts measured using two anthropomorphic phantoms, each phantom with six different isocenter locations and after five measurements (median and IQR)

Table S2 Distribution of the variation of the optical/thermal imaging positioning values during the delivery of a VMAT plan, for a cold and a warm surface (median and IQR)

Table S3 Four treatment couch displacements, in the lateral (*X*) and longitudinal (*Y*) directions, and the difference between the positioning values reported by surface/thermal‐ and stereoscopic X‐ray imaging, respectively, and the distance between the MV radiation center and the BB's center, in a 2 × 2‐cm^2^ field (25 MU, 6‐MV photons)

Table S4 Residual difference (median and IQR) between IGRT‐aligned isocenter (BB's center) and MV radiation field center (median and IQR), in a 2 × 2‐cm^2^ field (25 MU, 6‐MV photons). Results representing 12 IGRT‐positioned phantom exposures
