# Supplementary figures and images for: ExacTrac Dynamic workflow evaluation: Combined surface optical/thermal imaging and X‐ray positioning
Source: J Appl Clin Med Phys. 2022 Aug 24;23(10):e13754. doi: 10.1002/acm2.13754 (PMC9588276; doi:10.1002/acm2.13754)

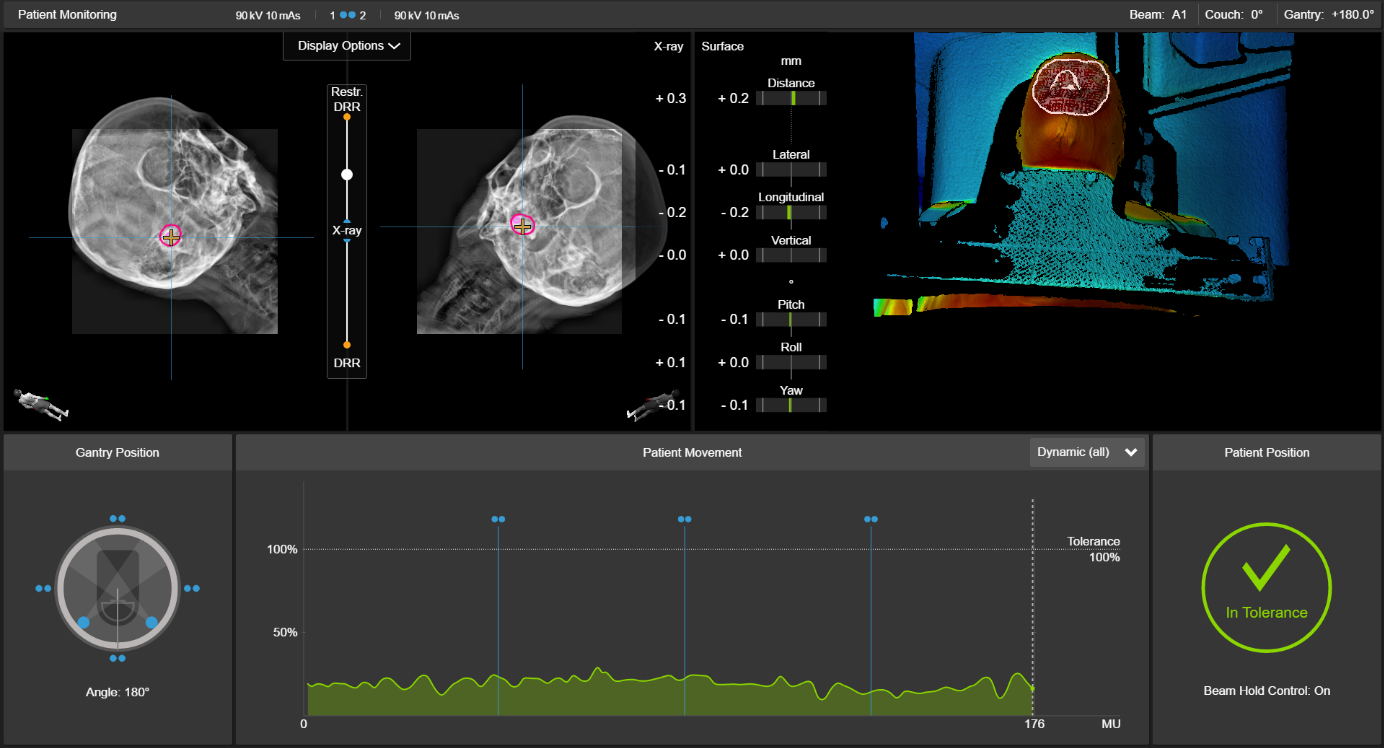

Supplement: Supplementary file 1 — FigureS1 [file ACM2-23-e13754-s001.png]

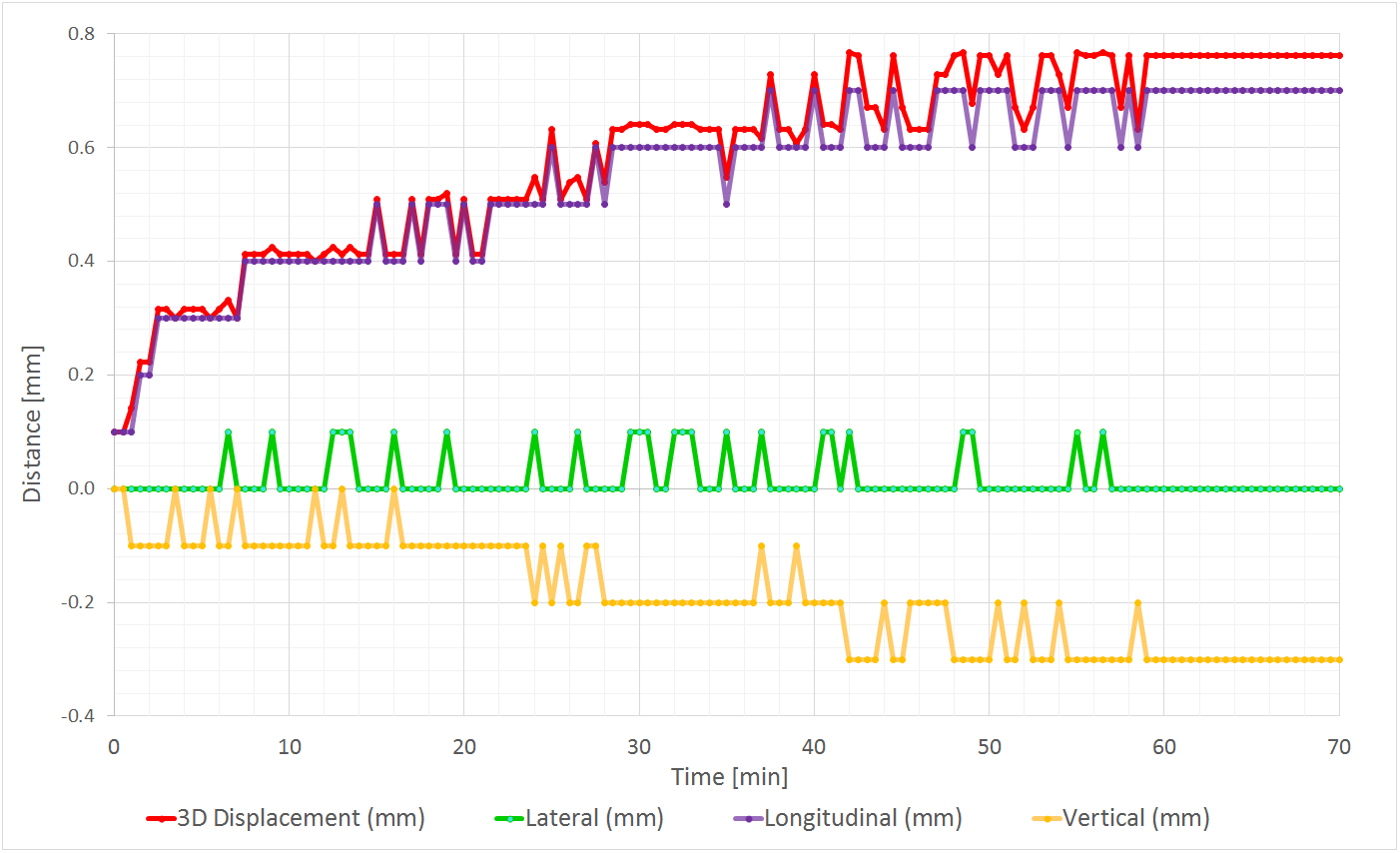

Supplement: Supplementary file 2 — FigureS2 [file ACM2-23-e13754-s002.png]

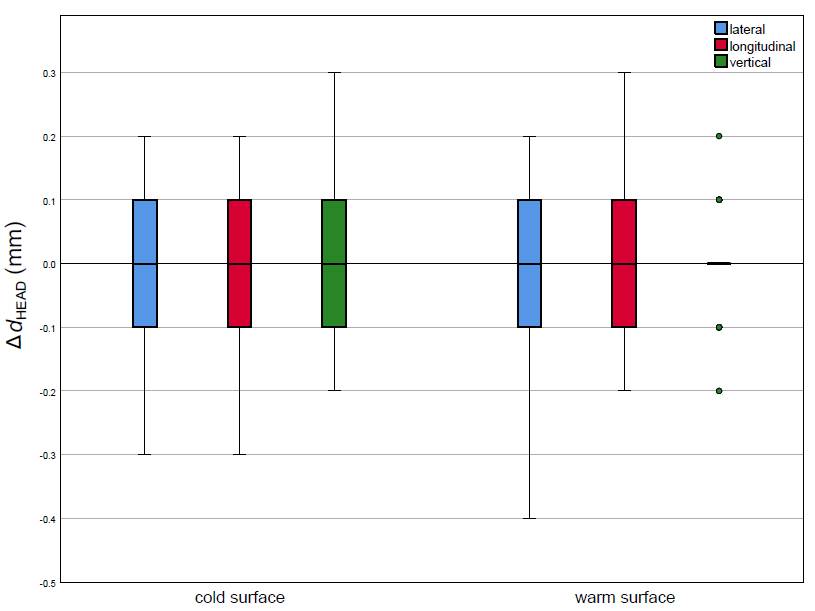

Supplement: Supplementary file 3 — FigureS3 [file ACM2-23-e13754-s009.png]

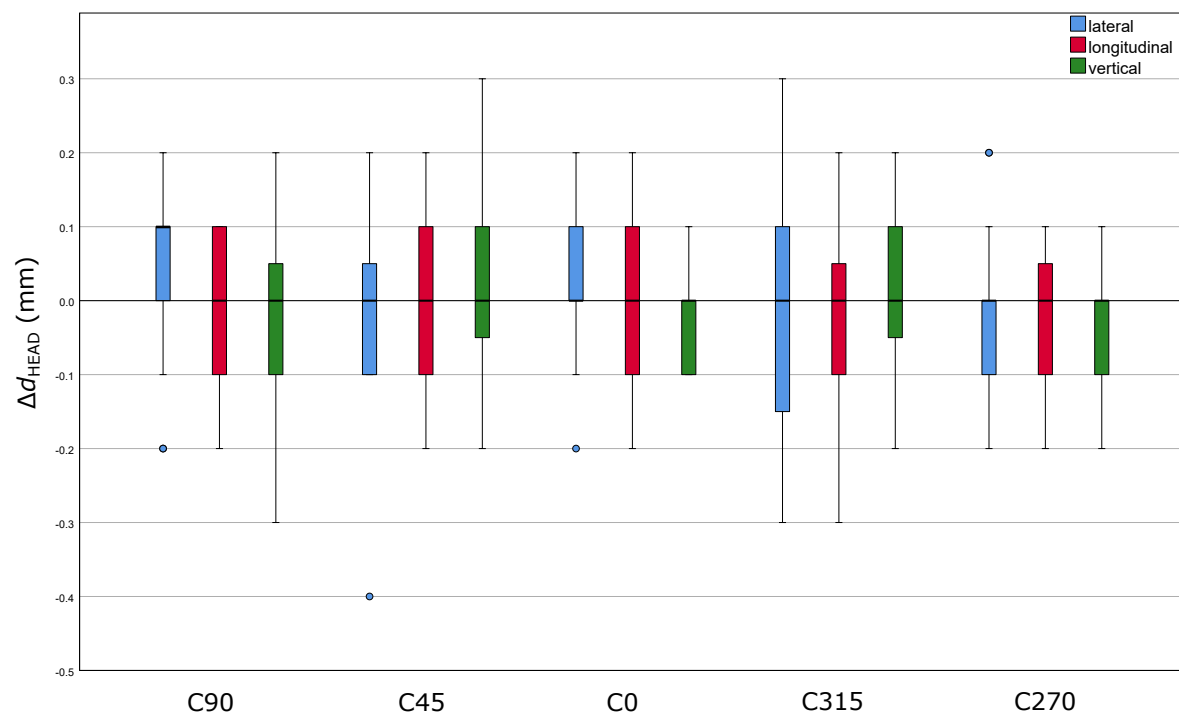

Supplement: Supplementary file 4 — FigureS4 [file ACM2-23-e13754-s004.pdf]
