# Supplementary material for: ExacTrac Dynamic workflow evaluation: Combined surface optical/thermal imaging and X‐ray positioning
Source: J Appl Clin Med Phys. 2022 Aug 24;23(10):e13754. doi: 10.1002/acm2.13754 (PMC9588276; doi:10.1002/acm2.13754)
Supplement: Supplementary file 5 — Figure S1 Patient monitoring. Top left: stereoscopic X‐ray imaging providing information about the internal bony structures. Top right: optical/thermal imaging of the AOI. In the center, each imaging system indicates the difference between the actual and the planned position in all 6 DoF. Bottom: Graphical representation of the intra‐fractional motion during the treatment [file ACM2-23-e13754-s006.docx]

Figure S1: Patient monitoring. Top left: stereoscopic X-ray imaging providing information about the internal bony structures. Top right: optical/thermal imaging of the AOI. In the centre, each imaging system indicate the difference between the actual and the planned position in all 6 DoF. Bottom: Graphical representation of the intra-fractional motion during the treatment.
