# Supplementary material for: ExacTrac Dynamic workflow evaluation: Combined surface optical/thermal imaging and X‐ray positioning
Source: J Appl Clin Med Phys. 2022 Aug 24;23(10):e13754. doi: 10.1002/acm2.13754 (PMC9588276; doi:10.1002/acm2.13754)
Supplement: Supplementary file 6 — Figure S2 Deviations recorded from the initial position in the lateral, longitudinal, and vertical directions, as well as the 3D displacement vector of the total deviation, for a cold surface [file ACM2-23-e13754-s012.docx]

Figure S2: Deviations recorded from the initial position in the lateral, longitudinal and vertical directions, as well as the 3D displacement vector of the total deviation, for a cold surface.
