# Supplementary material for: ExacTrac Dynamic workflow evaluation: Combined surface optical/thermal imaging and X‐ray positioning
Source: J Appl Clin Med Phys. 2022 Aug 24;23(10):e13754. doi: 10.1002/acm2.13754 (PMC9588276; doi:10.1002/acm2.13754)
Supplement: Supplementary file 7 — Figure S3 Boxplots of the distribution of the differences between optical/thermal‐ and X‐ray imaging positioning values (25 displacements at couch 0°, 4 repetitions) for a cold and a warm surface of the head phantom. The results for the different directions, lateral, longitudinal, and vertical, are plotted in blue, red, and green, respectively. The boxplots indicate the spread of the central 50% of the data, denominated as IQR. The median, the 25th (Q1) and the 75th (Q3) percentiles are also shown. The upper and the lower whiskers represent data outside the IQR but inside the range defined by 1.5 × IQR. Outliers are defined as values outside the whiskers’ range. [file ACM2-23-e13754-s010.docx]

Figure S3: Boxplots of the distribution of the differences between optical/thermal- and X-ray imaging positioning values (25 displacements at couch 0°, 4 repetitions) for a cold and a warm surface of the head phantom. The results for the different directions, lateral, longitudinal and vertical, are plotted in blue, red and green, respectively. The boxplots indicate the spread of the central 50% of the data, denominated as IQR. The median, the 25^th^ (Q_1_) and the 75^th^ (Q_3_) percentiles are also shown. The upper and the lower whiskers represent data outside the IQR but inside the range defined by 1.5 × IQR. Outliers are defined as values outside the whiskers’ range.
