# Supplementary material for: ExacTrac Dynamic workflow evaluation: Combined surface optical/thermal imaging and X‐ray positioning
Source: J Appl Clin Med Phys. 2022 Aug 24;23(10):e13754. doi: 10.1002/acm2.13754 (PMC9588276; doi:10.1002/acm2.13754)
Supplement: Supplementary file 9 — Table S1 The difference between the stereoscopic X‐ray and CBCT positional shifts measured using two anthropomorphic phantoms, each phantom with six different isocenter locations and after five measurements (median and IQR) [file ACM2-23-e13754-s011.docx]

Table S1: The difference between the stereoscopic X-ray and CBCT positional shifts measured using 2 anthropomorphic phantoms, each phantom with 6 different isocentre locations and after 5 measurements (median and IQR).

| Stereoscopic X-ray imaging vs. CBCT | | | | | | |
| --- | --- | --- | --- | --- | --- | --- |
|  | *d*_CBCT-Xray,X_ (mm) | Δ*d*_CBCT-Xray,Y_ (mm) | Δ*d*_CBCT-Xray,Z_ (mm) | Δ*d*_CBCT-Xray,PITCH_  (°) | Δ*d*_CBCT-Xray,ROLL_ (°) | Δ*d*_CBCT-Xray,YAW_ (°) |
| Cranial verification phantom | -0.3  [-0.3; -0.2] | 0.4  [0.2; 0.4] | 0  [-0.2; 0.2] | 0.1  [0.1; 0.2] | 0.1  [-0.2; 0.2] | 0.5  [0.5; 0.6] |
| Pelvic verification phantom | -0.4  [-0.6; -0.3] | 0.3  [0; 0.6] | 0.1  [0; 0.2] | -0.3  [-0.9; 0.7] | -0.1  [-0.3; 0.1] | 0.8  [0.4; 0.1] |
