# Supplementary material for: ExacTrac Dynamic workflow evaluation: Combined surface optical/thermal imaging and X‐ray positioning
Source: J Appl Clin Med Phys. 2022 Aug 24;23(10):e13754. doi: 10.1002/acm2.13754 (PMC9588276; doi:10.1002/acm2.13754)
Supplement: Supplementary file 10 — Table S2 Distribution of the variation of the optical/thermal imaging positioning values during the delivery of a VMAT plan, for a cold and a warm surface (median and IQR) [file ACM2-23-e13754-s005.docx]

Table S2: Distribution of the variation of the optical/thermal imaging positioning values during the delivery of a VMAT plan, for a cold and a warm surface (median and IQR).

| Surface/Thermal imaging | | | | | | |
| --- | --- | --- | --- | --- | --- | --- |
|  | *d*_ST,X_ (mm) | *d*_ST,Y_ (mm) | *d*_ST,Z_ (mm) | *d*_ST,PITCH_ (°) | *d*_ST,ROLL_ (°) | *d*_ST,YAW_ (°) |
| Cold surface (mm) | 0.03  [-0.02; 0.10] | 0  [-0.04; 0.04] | 0.02  [-0.02; 0.07] | 0  [-0.01; 0.01] | -0.03  [-0.06; 0] | 0.02  [0; 0.04] |
| Warm surface (mm) | 0.07  [0.02; 0.13] | -0.01  [-0.05; 0.05] | -0.04  [-0.06; 0] | 0  [-0.02; 0.01] | -0.03  [-0.06; 0] | -0.02  [-0.03; -0.01] |
