# Supplementary material for: ExacTrac Dynamic workflow evaluation: Combined surface optical/thermal imaging and X‐ray positioning
Source: J Appl Clin Med Phys. 2022 Aug 24;23(10):e13754. doi: 10.1002/acm2.13754 (PMC9588276; doi:10.1002/acm2.13754)
Supplement: Supplementary file 11 — Table S3 Four treatment couch displacements, in the lateral (X) and longitudinal (Y) directions, and the difference between the positioning values reported by surface/thermal‐ and stereoscopic X‐ray imaging, respectively, and the distance between the MV radiation center and the BB's center, in a 2 × 2‐cm2 field (25 MU, 6‐MV photons) [file ACM2-23-e13754-s008.docx]

Table S3: Four treatment couch displacements, in the lateral (x) and longitudinal (y) directions, and the difference between the positioning values reported by surface/thermal- and stereoscopic X-ray imaging, respectively, and the distance between the MV radiation centre and the BB’s centre, in a 2×2 cm^2^ field (25 MU, 6MV photons).

| Couch angle | Couch displacement (mm) | | Surface/thermal imaging vs. MV portal image (mm) | | | Stereoscopic X-ray imaging vs. MV portal image (mm) | | |
| --- | --- | --- | --- | --- | --- | --- | --- | --- |
|  | s_x_ | s_y_ | Δ*d*_MV-IGRT,ST,X_ | Δ*d*_MV-IGRT,ST,Y_ | 3D displacement | Δ*d*_MV-IGRT,Xray,X_ | Δ*d*_MV-IGRT,Xray,Y_ | 3D displacement |
| 90° | 2.0 | 0.0 | 0.3 | 0.1 | 0.3 | 0.1 | 0.3 | 0.1 |
|  | 0.0 | 2.0 | 0.2 | 0.5 | 0.5 | 0.2 | 0.5 | 0.5 |
|  | 2.0 | 2.0 | 0.2 | 0.5 | 0.5 | 0 | 0.5 | 0.4 |
|  | -2.0 | -2.0 | -0.1 | 0 | 0 | 0 | 0 | 0 |
| 45° | 2.0 | 0.0 | 0.2 | 0.2 | 0.2 | 0.3 | 0.3 | 0.3 |
|  | 0.0 | 2.0 | -0.4 | 0.4 | 0.3 | -0.3 | 0.5 | 0.5 |
|  | 2.0 | 2.0 | -0.1 | 0.2 | 0 | -0.1 | 0.3 | 0.1 |
|  | -2.0 | -2.0 | -0.4 | 0 | 0.3 | -0.3 | 0.1 | 0.1 |
| 0° | 2.0 | 0.0 | 0.4 | -0.1 | 0.4 | 0.3 | -0.1 | 0.3 |
|  | 0.0 | 2.0 | 0.1 | -0.1 | 0.1 | 0 | 0.1 | 0.1 |
|  | 2.0 | 2.0 | 0.2 | 0 | 0.1 | 0.3 | 0 | 0.2 |
|  | -2.0 | -2.0 | 0.1 | -0.3 | 0.1 | 0 | -0.3 | 0.2 |
| 315° | 2.0 | 0.0 | 0.2 | 0 | 0.2 | 0.1 | 0.1 | 0.1 |
|  | 0.0 | 2.0 | 0.2 | 0.1 | 0.1 | 0.1 | 0.3 | 0.3 |
|  | 2.0 | 2.0 | 0.7 | 0.5 | 0.9 | 0.6 | 0.5 | 0.8 |
|  | -2.0 | -2.0 | 0 | 0 | 0 | -0.1 | 0 | 0.1 |
| 270° | 2.0 | 0.0 | 0.2 | 0 | 0.2 | 0.2 | 0.1 | 0.2 |
|  | 0.0 | 2.0 | 0 | 0.2 | 0.2 | 0.1 | 0.2 | 0.2 |
|  | 2.0 | 2.0 | 0.2 | 0.2 | 0.3 | 0.2 | 0.2 | 0.3 |
|  | -2.0 | -2.0 | 0.1 | -0.3 | 0.2 | 0 | -0.3 | 0.3 |
