# Supplementary material for: ExacTrac Dynamic workflow evaluation: Combined surface optical/thermal imaging and X‐ray positioning
Source: J Appl Clin Med Phys. 2022 Aug 24;23(10):e13754. doi: 10.1002/acm2.13754 (PMC9588276; doi:10.1002/acm2.13754)
Supplement: Supplementary file 12 — Table S4 Residual difference (median and IQR) between IGRT‐aligned isocenter (BB's center) and MV radiation field center (median and IQR), in a 2 × 2‐cm2 field (25 MU, 6‐MV photons). Results representing 12 IGRT‐positioned phantom exposures [file ACM2-23-e13754-s003.docx]

Table S4: Residual difference (median and IQR) between IGRT-aligned isocentre (BB’s centre) and MV radiation field centre (median and IQR), in a 2×2 cm^2^ field (25 MU, 6MV photons). Results representing 12 IGRT-positioned phantom exposures.

| Gantry Angle | | | | | | | | | |
| --- | --- | --- | --- | --- | --- | --- | --- | --- | --- |
|  | | 0° | | 90° | | 180° | | 270° | |
|  | Δ*d*_WL,X_ (mm) | | Δ*d*_WL,Y_ (mm) | Δ*d*_WL,Z_ (mm) | Δ*d*_WL,Y_ (mm) | Δ*d*_WL,X_ (mm) | Δ*d*_WL,Y_ (mm) | Δ*d*_WL,Z_ (mm) | Δ*d*_WL,Y_ (mm) |
| Median | 0.2 | | 0.5 | 0.5 | 0.2 | 0.5 | -0.7 | 0.2 | 0 |
| IQR | [0.2; 0.5] | | [0.5; 0.7] | [0.2; 0.5] | [0; 0.5] | [0.2; 0.5] | [-0.7; -0.5] | [0; 0.2] | [0; 0.2] |
